# Data for the evaluation of irrigation development interventions in Northern Ethiopia

**DOI:** 10.1016/j.dib.2019.104342

**Published:** 2019-08-01

**Authors:** Negusse Yigzaw, John Mburu, Chris Ackello Ogutu, Cory Whitney, Eike Luedeling

**Affiliations:** a World Agroforestry Centre (ICRAF), United Nations Avenue, Gigiri, P. O. Box 30677-00100, Nairobi, Kenya; b Center for Development Research (ZEF), University of Bonn, Genscherallee 3, D-53113, Bonn, Germany; c University of Nairobi, Dept. of Agricultural Economics, Kangemi, P. O. Box 29053-00625, Nairobi, Kenya; d University of Bonn, INRES – Horticultural Sciences, Auf Dem Hügel 6, D-53121, Bonn, Germany

**Keywords:** Ex-ante impact assessment, Feasibility study, Decision support, Cost benefit analysis, Water harvesting, Dam construction

## Abstract

This data article provides the datasets that are used in the holistic ex-ante impact evaluation of an irrigation dam construction project in Northern Ethiopia [1]. We used an expert knowledge elicitation approach as a means of acquiring the data. The data shared here captures all the parameters considered important in the impact pathway (i.e. the expected benefits, costs, and risks) of the decision to construct an irrigation dam. The dataset is disaggregated for two impact pathway models: one complementing the dam construction with catchment restoration and the other without catchment restoration. Both models are scripted in the R programming language. The data can be used to examine how the construction of an irrigation dam affects the incomes as well as the food and nutritional status of farmers that are affected by the intervention.

**Table:** Specifications Table

Subject	Environmental science
Specific subject area	Ecological Modelling, Water Science and Technology, Management, Monitoring, Policy and Low
Type of data	Table Graph Code, scripted in R-programming language
How data were acquired	The data were acquired from experts
Data format	Raw Analyzed (i.e. model generated)
Parameters for data collection	Experts who are knowledgeable about the dam construction were selected purposively. Accordingly, their knowledge about the embankment dam, the construction site and familiarity with the planned intervention were the main criteria for selecting them. To capture the detailed impact pathway of the intervention, the experts were selected from different fields of specialization.
Description of data collection	The experts developed an impact pathway model that shows the expected costs, benefits and risks of the proposed intervention. Before collecting the quantitative estimates, the experts were calibrated informally. Quantitative data was collected based on the experts' state of understanding.
Data source location	City/Town/Region: Ebo village, Raya-Azebo Woreda (district), Tigray Region Country: Ethiopia Latitude and longitude for collected samples/data: 39.63°–39.66° E and 12.86°–12.89° N
Data accessibility	The data are hosted with this article
Related research article	N. Yigzaw, J. Mburu, C. Ackello-Ogutu, C. Whitney, E. Luedeling, Stochastic impact evaluation of an irrigation development intervention in Northern Ethiopia. Sci. Total Environ. 685 (2019) 1209-1220. https://doi.org/10.1016/j.scitotenv.2019.06.133

**Table:** 

**Value of the Data** • The data are used in the *ex-ante* evaluation of an irrigation dam. • The data provide insights for investigating the welfare impacts of irrigation dams (both *with* and *without* catchment restoration practices). • The data can be used to examine how the construction of an irrigation dam affects income as well as food and nutritional status of farmers that are affected by the intervention. • The data are valuable for holistic impact evaluation that captures important ecological and socio-economic variables. • The data may be useful for future researcher on the impacts of investing in irrigation dams.

## 1 Data

The data are used in the holistic ex-ante impact evaluation of an irrigation dam in northern Ethiopia. The data captured all important impact parameters (i.e. expected benefits, costs, and risks) of the proposed dam. The impact model is disaggregated by intervention design, with impacts on various stakeholders captured separately. Our simulations cover economic, ecological and nutritional impacts of the intervention.

This article presents three data sets:

a) Input data used to run the simulation model. These consist of three.csv datasets:
  • an estimate sheet, which contains the data elicited from experts (dam_estimates.csv),
  • a correlation matrix that defines relationships for correlated parameters (dam_estimates_cor.csv) and
  • a legend table that translates variable names into readable figure labels (dam_legend.csv).
b) Simulation model script (dam_code.r) written in the R programming language [2] using the decisionSupport package [3].
c) The output results of the simulation are presented as spreadsheets (mcSimulationResults.csv) and graphs (contents of the ‘compound figures’ folder).

## 2 Experimental design, materials, and methods

### 2.1 Model development

To develop an impact pathway model and collect quantitative estimates of the input parameters, we followed a decision analysis approach [4]. The approach seeks to actively involve local experts in development of both an impact pathway and a final decision model. In this case, ‘experts’ refers to local knowledge holders with in-depth understanding of the local context and the potential benefits, costs and risks of dam construction.

Model development began with an interview with the head of the region's irrigation development program, the official who would be the coordinator of the intended project. This coordinator helped to identify ten experts with knowledge of similar local systems. These ten experts were asked to consider the dam construction project decision and identify the main effects of the proposed project, which they grouped into factors of relevance to the local community (hereafter referred to as stakeholders), the environment, and the implementer. Based on location and access to the irrigation scheme (i.e. the land with access to water from the irrigation project), the stakeholders were further classified into four groups: (i) downstream irrigators (farmers who would use the water from the dam for agriculture in an irrigation scheme), (ii) downstream non-irrigators (farmers downstream who could be affected by the dam but do not have access to the irrigation water), (iii) displaced farmers (those who currently live in the dam construction area but will need to be relocated and compensated, both financially and with a parcel of land in the irrigation scheme), and (iv) upstream non-irrigators (those who live upstream of the dam, close enough to access water for domestic use and/or livestock consumption).

We generated a preliminary qualitative impact pathway model based on local expert knowledge, including important costs, benefits and risks of the project for the stakeholders, the implementer and the environment. This preliminary model was then shared with the experts for review and refined based on their feedback. Moreover, the identified environmental impact estimates were further refined based on existing literature. We consolidated all inputs into a final model capturing the impact pathway of the proposed intervention, which reflected the current state of understanding and knowledge of all experts [4].

### 2.2 Model parametrization

Experts provided quantitative estimates for all model variables covering the benefits, costs and risks of the dam construction. We used an expert knowledge elicitation approach, which is also used in other studies [4], [5], [6], to collect estimates for model variables. All experts were familiarized with estimation techniques to overcome bias, allowing them to provide quantitative estimates in the form of distributions representing their subjective 90% confidence intervals [7]. Initial monetary estimates were collected in Ethiopian currency (i.e. Birr) and converted to USD at the Ethiopian National Bank's October 10, 2017 rate of $1 = 23.3908 ETB [8].

### 2.3 Data analysis

The resulting model was scripted in the R programming language [2] and run 10,000 times as a Monte Carlo simulation to randomly select values from defined distributions for all input variables [9] using the ‘decisionSupport’ package [3]. The intervention was modeled in two ways: 1) the dam construction is complemented with catchment restoration; 2) the dam construction is implemented without catchment restoration. The model was used to provide forecasts of the costs, benefits and risks of the irrigation project for each stakeholder, the implementer, and the environment. The forecasted marginal benefits/losses were discounted according to an estimated discount rate to compute the monetized net benefit, expressed as net present value (NPV), representing the sum of discounted projected net benefits over the expected life span of the project [4]. More information about the computation of the marginal benefits/losses is provided in the supplementary material (Appendix A). In order to guide the decision, we also computed the total project outcome (i.e. the sum of NPVs for all stakeholders, the implementer and the environment). Additional information about nutritional outcomes is provided in Yigzaw et al. [1].
