# Supplementary material for: Data for the evaluation of irrigation development interventions in Northern Ethiopia
Source: Data Brief. 2019 Aug 1;25:104342. doi: 10.1016/j.dib.2019.104342 (PMC6717167; doi:10.1016/j.dib.2019.104342)
Supplement: Multimedia component 1 [file mmc1.docx]

# **Supplemental Information:**

# **Stochastic Impact Evaluation of an Irrigation Development Intervention in Northern Ethiopia**

Negusse Yigzaw^1,2,3^, Cory Whitney^2,3^, Chris-Ackello Ogutu^3^, John Mburu^3^, Eike Luedeling^1,4^

^1^ World Agroforestry Centre (ICRAF), United Nations Avenue, Gigiri, P. O. Box 30677-00100, Nairobi, Kenya

^2^ Center for Development Research (ZEF), University of Bonn, Genscherallee 3, D-53113, Bonn, Germany

^3^ University of Nairobi, Dept. of Agricultural Economics, Kangemi, P. O. Box 29053-00625, Nairobi, Kenya

^4^ University of Bonn, Department of Horticultural Sciences, Auf dem Hügel 6, D-53121, Bonn, Germany

Correspondence: Negusse Yigzaw, email. [N.Yigzaw@cgiar.org](mailto:N.Yigzaw@cgiar.org) or [negusseyigzaw@gmail.com](mailto:negusseyigzaw@gmail.com)

# **Methodology**

**Decision modelling**

The model computes the marginal benefits and losses of implementing a dam project by subtracting the total expected costs from the expected benefit. The expected total benefit is the sum of all additional benefits, such as crop production and employment opportunities, obtained from the implementation of the project (i.e. with project) and the benefit of reducing losses or damages, like flooding effect, by having the dam (i.e. the reduction of costs incurred under ‘the business as usual scenario’). The total expected cost is also computed by adding up all the additional costs incurred by having the dam, such as agricultural production costs, costs of displacement, project costs (i.e. with project), and the benefit forgone (under the ‘business as usual’) by having the structure, including the productivity lost and negative environmental effects.

The study used the decision analysis tools: Monte Carlo simulation, Partial Least Squares regression, and Value of Information analysis (Luedeling et al., 2015; Whitney et al., 2017) to project the outcome of the proposed intervention, we identify the sensitive variables that influence the distribution of the decision outcomes, as well as critical knowledge gaps that may change the emerging decision recommendation.

***Monte Carlo Simulation***

Monte Carlo simulation works by randomly selecting values from the specified range of values, which follows a predefined distribution of the parameter, and computes the expected output deterministically. This action is repeated (i.e. 10,000 times in this study), to generate an aggregated outcome. The difference between the upper and lower limit in each range of value indicates our (analysts and experts) level of calibrated uncertainty for the specified parameter.

NPV represents the sum of discounted projected net benefits over the expected life span of the project. The NPV was computed for all the stakeholders, the environment and the implementer, and these NPVs were aggregated to compute the overall outcome of the proposed irrigation dam project. Mathematically the total project NPV is;

$$NPV=\sum_{i=1}^{6} \sum_{t=1}^{30} \frac{C_{it}}{{(1+r)}^{t}}$$

Where, *i* is the expected group to be affected by the proposed intervention (e.g. *i*=1 for downstream farmers, 2 for displaced farmers), *t* is the time of the cash flow, *r* is the discount rate used, and *C_it_* is the risk-adjusted net cash flow for the expected group *i* at time *t* (i.e. the difference between the risk-adjusted total benefits for the expected group *i* at time *t* and the risk-adjusted total costs for the expected group *i* at time *t*).

The nutritional (i.e. energy and Vitamin A) outcomes of the proposed intervention were computed from the net additional production of the agricultural area, after considering post-harvest losses. The net agricultural production is the difference between the yield obtained from the irrigation scheme and the expected yield lost due to inundation of current farmland. The nutritional outcomes were expressed according to the number of people whose annual needs could be covered by the additional agricultural production expected within the proposed irrigation scheme. These were computed by dividing the total nutrient production by the annual per capita requirements.

***Partial Least Squares regression***

Partial Least Squares (PLS) regression identifies linear combinations of the input variables that explain variation in a dependent variable based on the principle of variable compression (Luedeling and Gassner, 2012). PLS was used to regress the NPV of the proposed project outcomes against the all input variables.

***Value of Information analysis***

We identified the variables with highest value of information (i.e. variables that most influence the sign of the project outcome) using Value of Information (VoI) analysis (Tuffaha et al., 2016). We used the expected value of perfect information (EVPI) procedure described by Wafula et al. (2018), to calculate the monetary value of additional information for the decision-making process. The EVPI is the difference between the expected outcome of the proposed intervention under perfect information and the expected outcome of implementing the proposed intervention under current information. Mathematically EVPI is;

$$EVPI= {EV}_{wPI}-{EV}_{woPI}$$

Where EVPI is the expected value of perfect information, ${EV}_{wPI}$ is the the expected value of a decision with perfect information, and ${EV}_{woPI}$ is the expected value of a decision without perfect information. EVPI indicates the maximum value that a decision maker should be willing to pay in order to reduce uncertainty about which decision alternative promises the greatest returns (Whitney et al., 2017)

# **Results**

### **Tables**

**Table S1**: List of important benefits and costs identified by the experts for the implementation of an irrigation dam project in Tigray, Ethiopia, and applicability to each stakeholder group, the environment, and the implementer.

| All identified impacts of the proposed dam construction intervention | Downstream irrigators | Downstream non-irrigators | Displaced farmers | Upstream non-irrigators | Environment | Implementers |
| --- | --- | --- | --- | --- | --- | --- |
| ***Benefits of the poposed project*** |  |  |  |  |  |  |
| - Dry season irrigation | Yes | – | Yes | – | – | – |
| - Supplementing rainfed agriculture during unexpected dry spells in the rainy season | Yes | – | Yes | – | – | – |
| - Employment generation | Yes | Yes | Yes | Yes | – | – |
| - Income from compensation payments | – | – | Yes | – | – | – |
| - Time savings (fetching water and watering livestock) | – | – | – | Yes | – | – |
| - Access to better infrastructure | – | – | – | Yes | – | – |
| - Flood control | Yes | Yes | – | – | – | – |
| - Ecosystem services from catchment restoration (provisioning, regulating and maintenance, and cultural services) | – | – | – | – | Yes | – |
| - Ecosystem services from restoring vegetation in the vicinity of the reservoir (provisioning, regulating and maintenance, and cultural services) | – | – | – | – | Yes | – |
| ***Costs of the proposed project*** |  |  |  |  |  |  |
| - Dam and infrastructure construction (including study, design, monitoring and supervision) | – | – | – | – | – | Yes |
| - Catchment restoration (soil conservation, reforestation, and area exclosure) | – | – | – | – | – | Yes |
| - Compensation (for farming plot, house and public infrastructure) | – | – | – | – | – | Yes |
| - Repair and maintenance | – | – | – | – | – | Yes |
| - Inputs for agricultural production | Yes | – | Yes | – | – | – |
| - Additional expenses (e.g. bills for utilities) for displaced farmers who pay for access to new infrastructure | – | – | Yes | – | – | – |
| - Residence construction (for displaced farmers) | – | – | Yes | – | – | – |
| - Social and cultural cost of displacement | – | – | Yes | – | – | – |
| - Loss of agricultural production during construction | – | – | Yes | – | – | – |
| - Yield reduction further downstream by holding water in the reservoir and catchment | – | Yes | – | – | – | – |
| - Reduction in alluvial deposits | Yes | Yes | Yes | – | – | – |
| - Value of farming area lost to the splitting up between settled and displaced farmers | Yes | – | – | – | – | – |
| - Ecosystem services forgone from the area under the dam | – | – | – | – | Yes | – |
| - Ecosystem services forgone from land degradation (i.e. reduction in vegetation, grass, and forest land) due to water storage | – | – | – | – | Yes | – |

*Yes* indicates the identified cost and/or benefit applies for the specific group, while *‘–‘* indicates that it does not.

**Table S2**: Classification of risks identified by the experts based on their expected effect on the selected benefits and costs of implementing an irrigation dam project in Tigray, Ethiopia

| Risks that reduce benefits | Risks that increase costs | Risks that lead to total project failure |
| --- | --- | --- |
| - Delay in construction time - Occurence of dry spell - Rainfall shortage (i.e. below the expected minimum threshold) - Flood water diversion into another farming area before it reaches the dam - Water abstraction by the municipality for supplying a nearby town - Water abstraction for domestic and livestock consumption by farmers - Reduction in sale price of farm products due to excess supply | - Construction delay - Increase in construction cost - Increase in malaria incidence - Sedimentation | - Dam failure |

## **Simulation results**

**Table S3**: Distribution of Net Present Value (NPV) based on 10,000 Monte Carlo model runs for all stakeholders, the implementer, the environment, and total project outcome for the implementation of an irrigation dam project, including catchment restoration in Tigray, Ethiopia.

| Description |  | Distribution of simulation outcome (Million USD) | | |  | Chance (%) | |
| --- | --- | --- | --- | --- | --- | --- | --- |
|  |  | 5% | 50% | 95% |  | loss | gain |
| Environment |  | 3.13 | 4.56 | 6.44 |  | 0.25 | 99.75 |
| Downstream irrigators |  | 1.39 | 3.62 | 6.16 |  | 2.26 | 97.74 |
| Displaced irrigators |  | -0.46 | 0.39 | 1.01 |  | 20.86 | 79.14 |
| Upstream non-irrigators |  | 0.14 | 0.29 | 0.53 |  | 0 | 100 |
| Downstream non-irrigators |  | -0.27 | 0.06 | 0.21 |  | 32.32 | 67.68 |
| Implementer |  | -17.13 | -13.18 | -10.67 |  | 100 | 0 |
| Total project effect |  | -9.69 | -4.33 | 0.34 |  | 93.77 | 6.23 |

*Note: the chance of loss is the percentage of total model runs in which the NPV is negative. Gain indicates the percentage of results with positive NPV.*

**Table S4**: Distribution of Net Present Value (NPV) based on 10,000 Monte Carlo model runs for all the stakeholders, the implementer, the environment, and total project outcome for the implementation of an irrigation dam project, without catchment restoration in Tigray, Ethiopia

| Description |  | Distribution of simulation outcome (Million USD) | | |  | Chance (%) | |
| --- | --- | --- | --- | --- | --- | --- | --- |
|  |  | 5% | 50% | 95% |  | loss | gain |
| Environment |  | -0.31 | -0.21 | -0.14 |  | 100 | 0 |
| Downstream irrigators |  | 1.25 | 3.35 | 5.74 |  | 2.42 | 97.58 |
| Displaced irrigators |  | -0.5 | 0.34 | 0.95 |  | 23.51 | 76.49 |
| Upstream non-irrigators |  | 0.14 | 0.29 | 0.52 |  | 0 | 100 |
| Downstream non-irrigators |  | -0.28 | 0.06 | 0.21 |  | 32.57 | 67.43 |
| Implementer |  | -17.25 | -13.29 | -10.76 |  | 100 | 0 |
| Total project effect |  | -14.56 | -9.53 | -5.72 |  | 100 | 0 |

*Note: the chance of loss is the percentage of total model runs in which the NPV is negative. Gain indicates the percentage of results with positive NPV.*

# **Figures:**

1. **Figures for the outcomes of the dam project without catchment restoration**


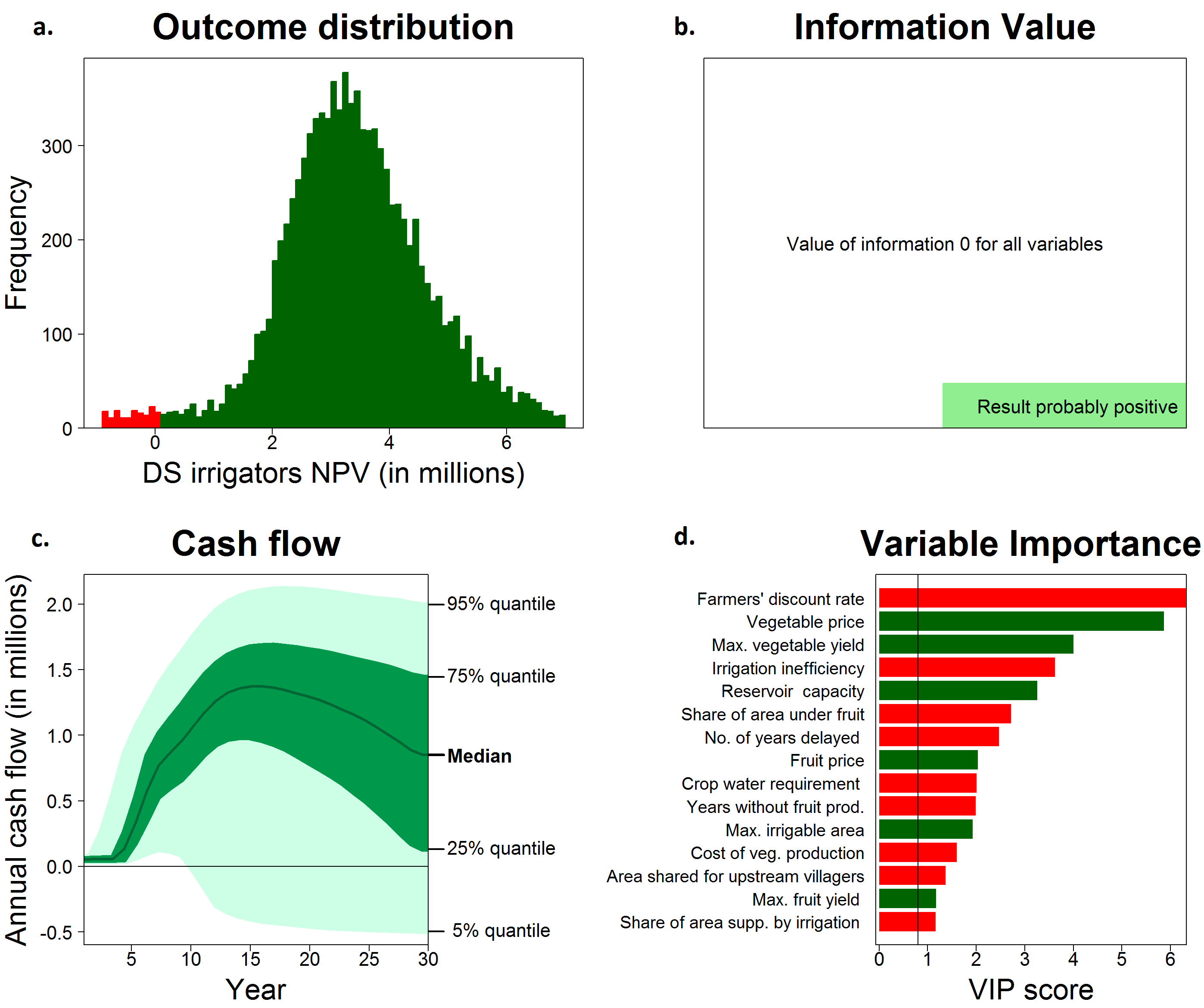


**Figure S1**: Simulation results from 10,000 model runs for the **downstream irrigators** for the implementation of a proposed irrigation dam, **without catchment restoration**, in Tigray, Ethiopia.

***a.*** *distribution of project outcome expressed as the Net Present Value (NPV), positive (green) and negative (red).* ***b.*** *Expected Value of Perfect Information (EVPI). EVPI>0 indicates that the selected uncertain variable has information value and further measurement is recommended to reduce model uncertainty.* ***c.*** *distribution of modeled annual net cashflow over the expected project life span of 30 years.* ***d.*** *variable importance in the projection (VIP) highlighting all model variables with a VIP greater than 0.8 (black vertical line) and correlation with project outcome, positive (green) and negative (red).*


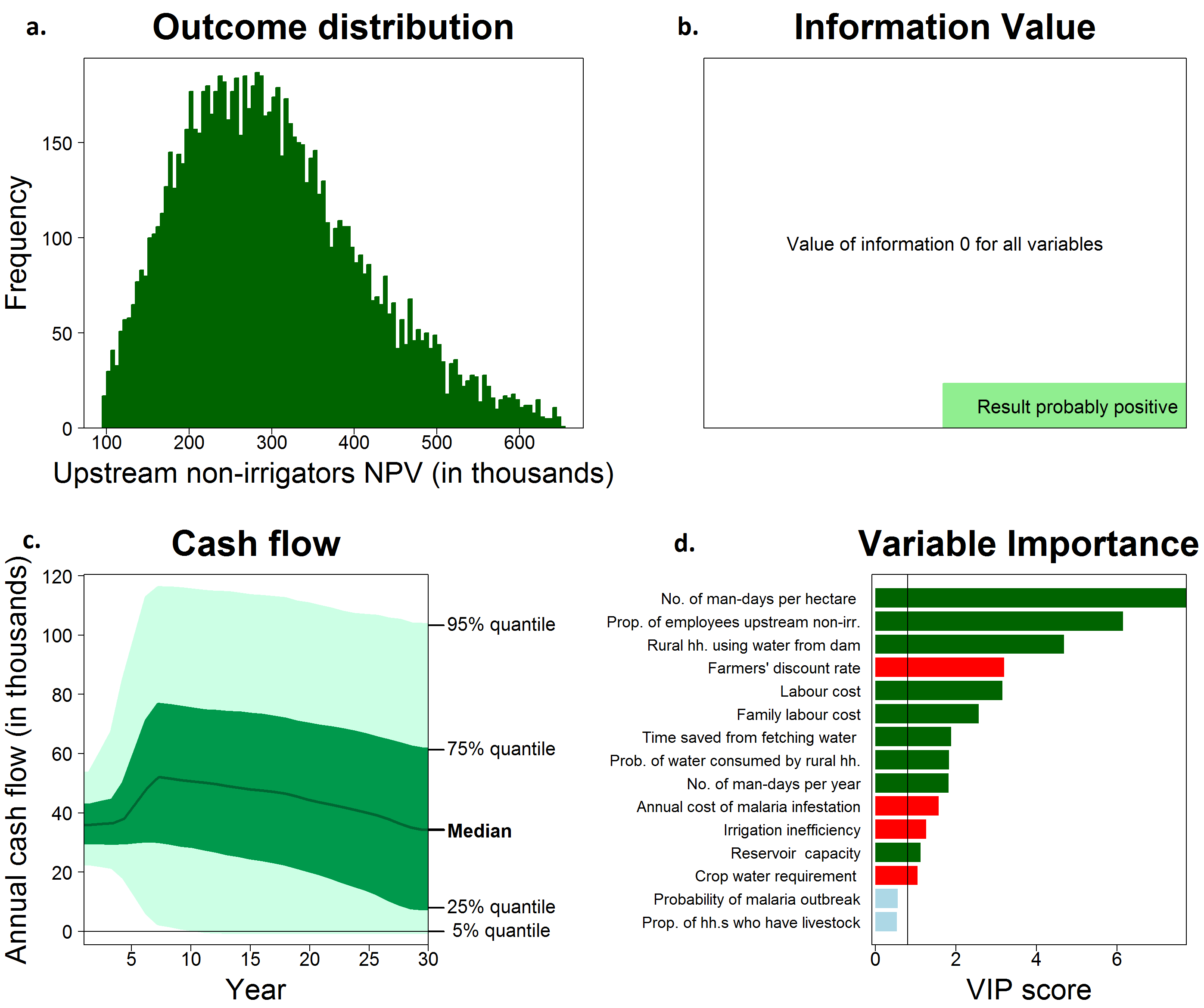


**Figure S2**: Simulation results from 10,000 model runs for the **upstream non-irrigators** for the implementation of a proposed irrigation dam, **without restoration**, in Tigray, Ethiopia.

For detailed description of the graphs and bars, see legend to Figure S1


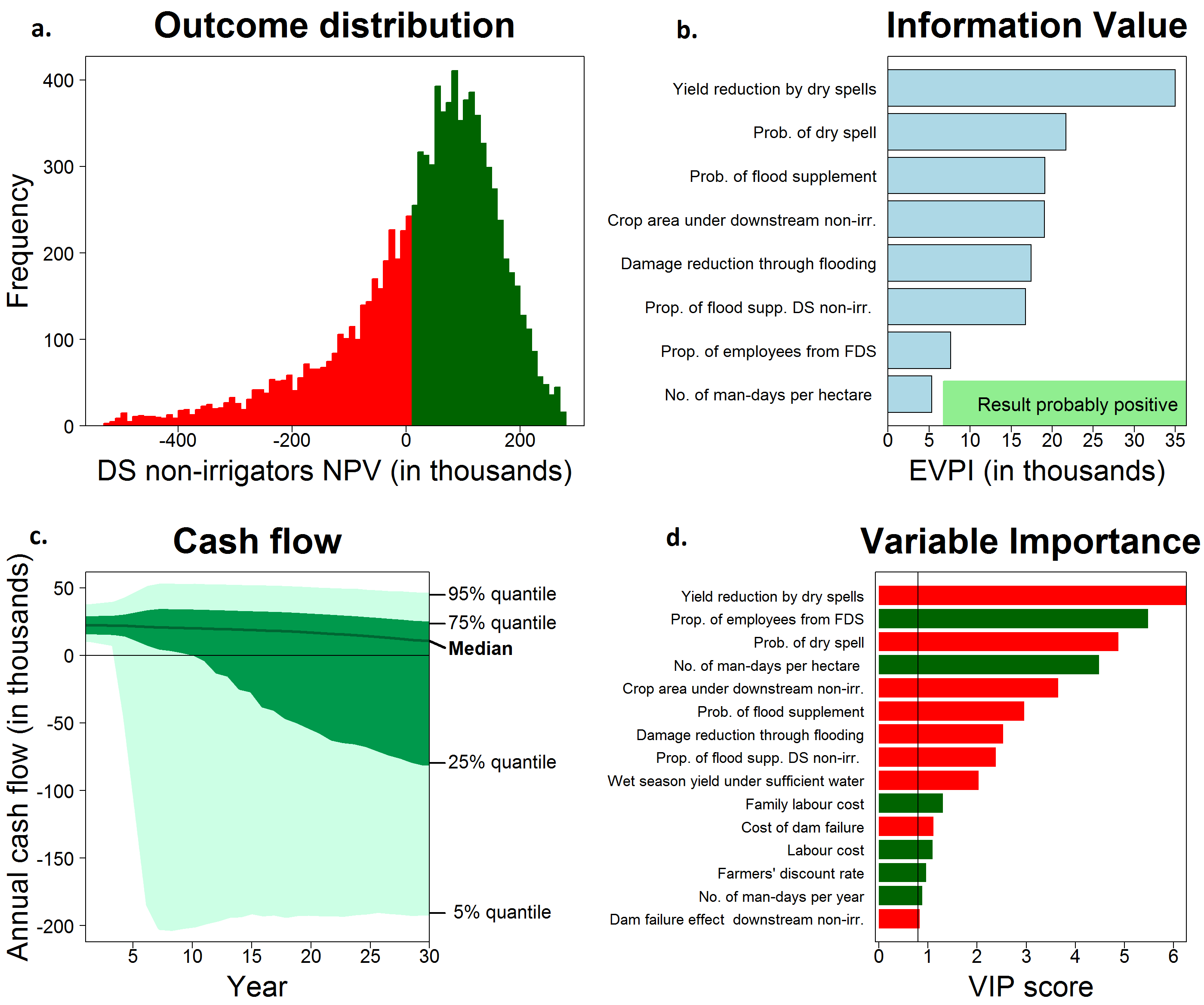


**Figure S3:** Simulation results from 10,000 model runs for the **downstream non-irrigators** for the implementation of a proposed irrigation dam, **without restoration**, in Tigray, Ethiopia.

For detailed description of the graphs and bars, see legend to Figure S1


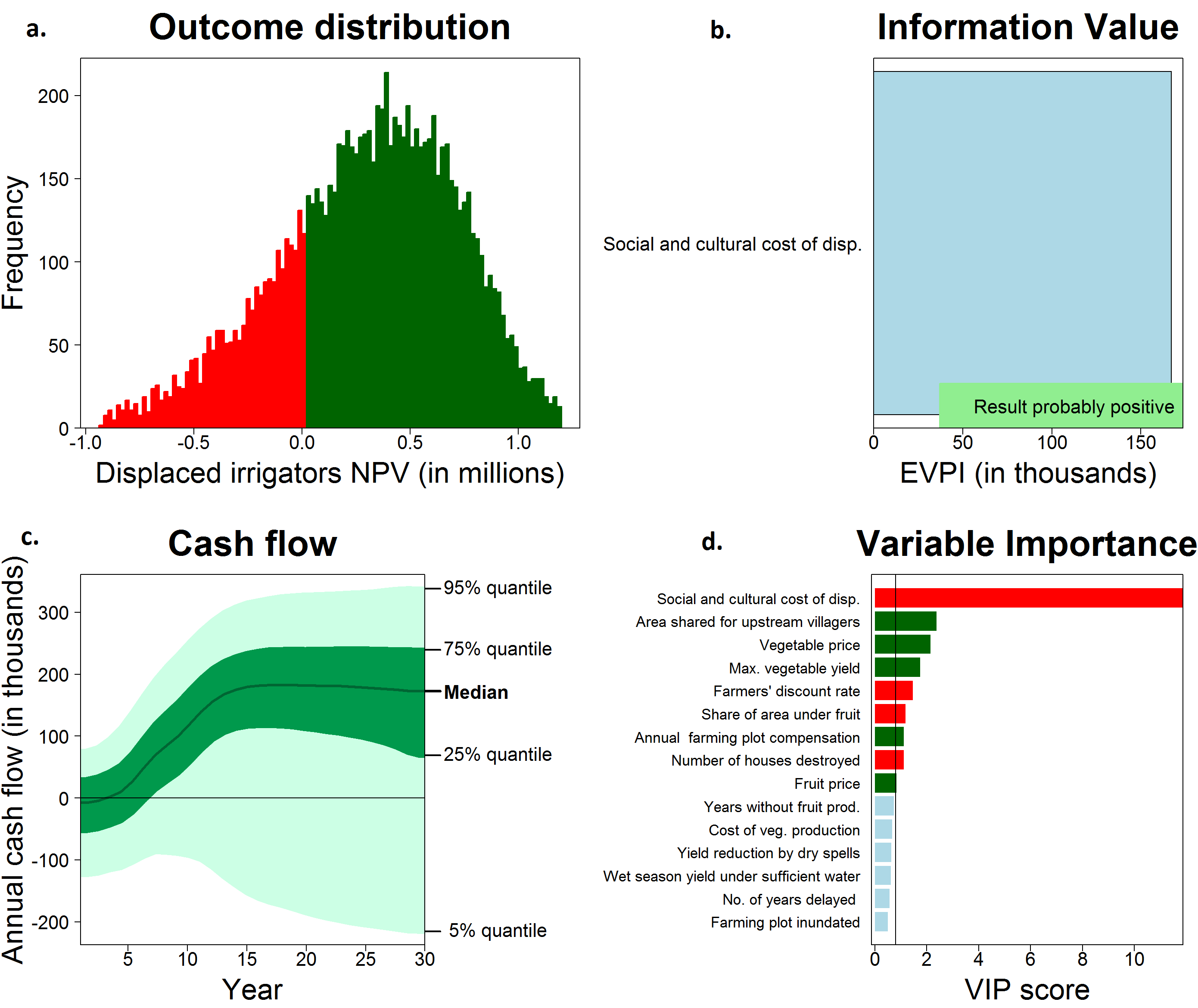


**Figure S4:** Simulation results from 10,000 model runs for the **displaced irrigators** for the implementation of a proposed irrigation dam, **without restoration**, in Tigray, Ethiopia.

For detailed description of the graphs and bars, see legend to Figure S1.


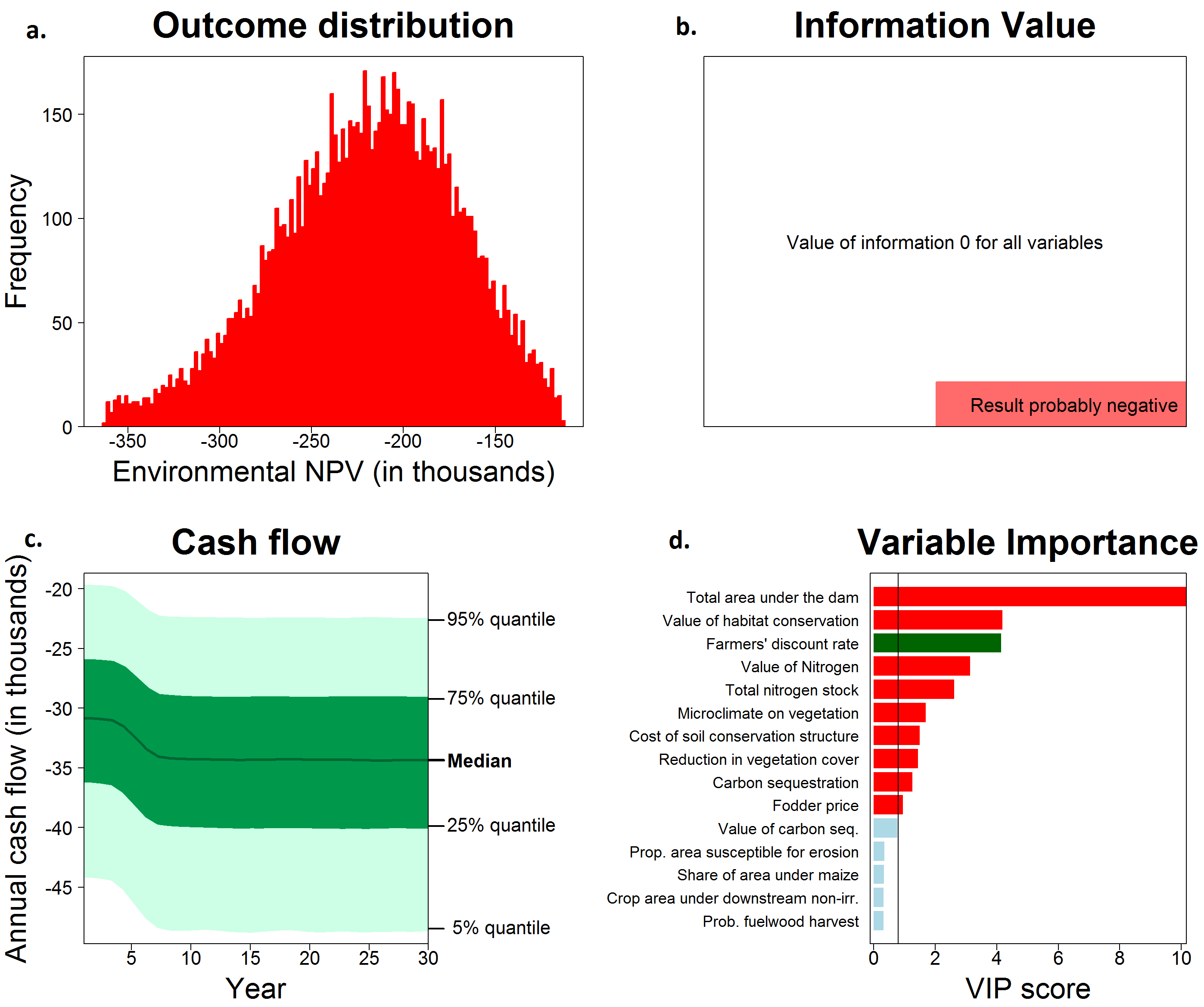


**Figure S5**: Simulation results from 10,000 model runs for the **environmental** **effect** of implementing an irrigation dam project, **without restoration**, in Tigray, Ethiopia.

For detailed description of the graphs and bars, see legend to Figure S1


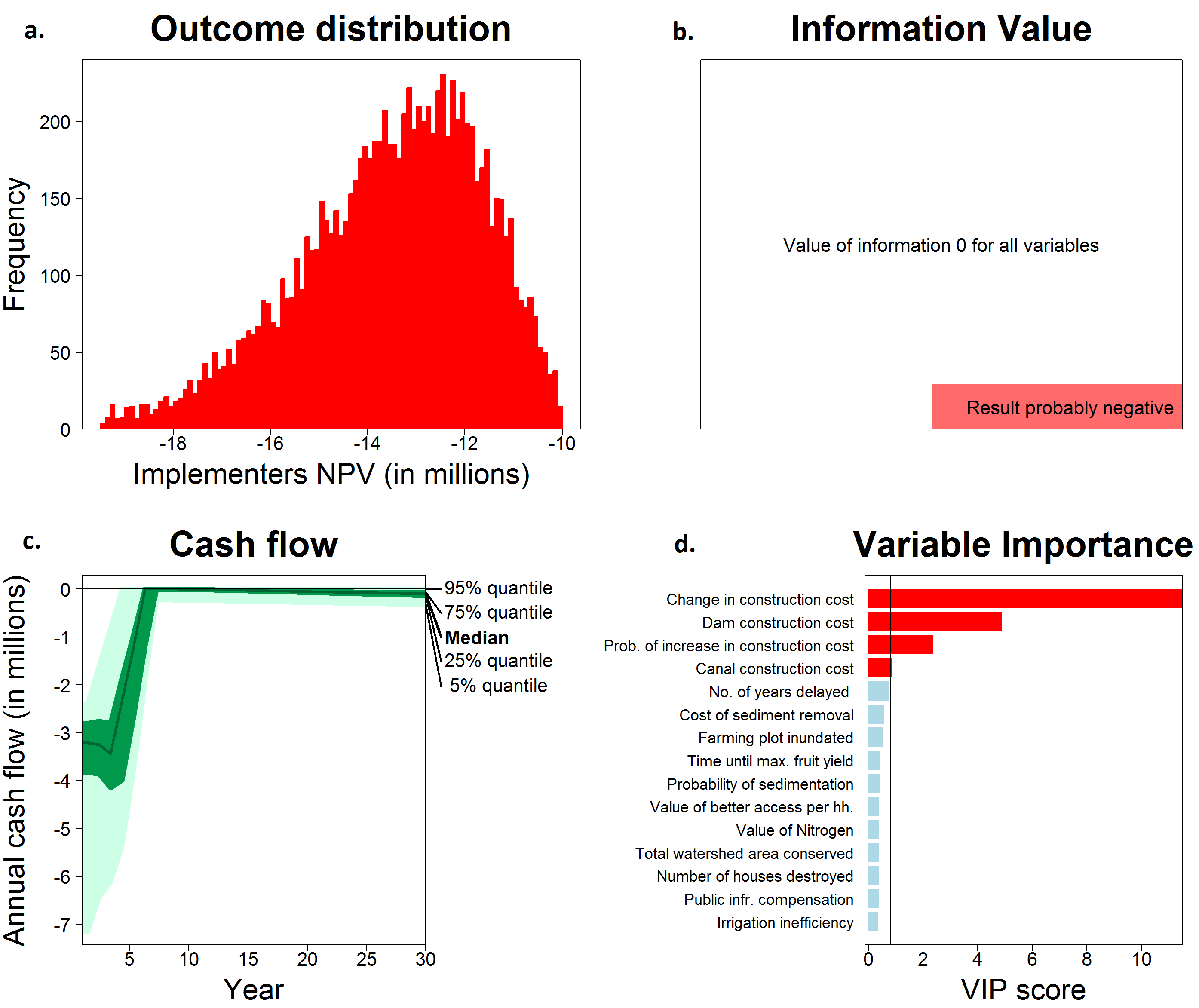


**Figure S6**: Simulation results from 10,000 model runs for the **implementer** for the implementation of a proposed irrigation dam, **without restoration**, in Tigray, Ethiopia.

For detailed description of the graphs and bars, see legend to Figure S1

1. **Figures for the outcomes of the damming with catchment restoration**


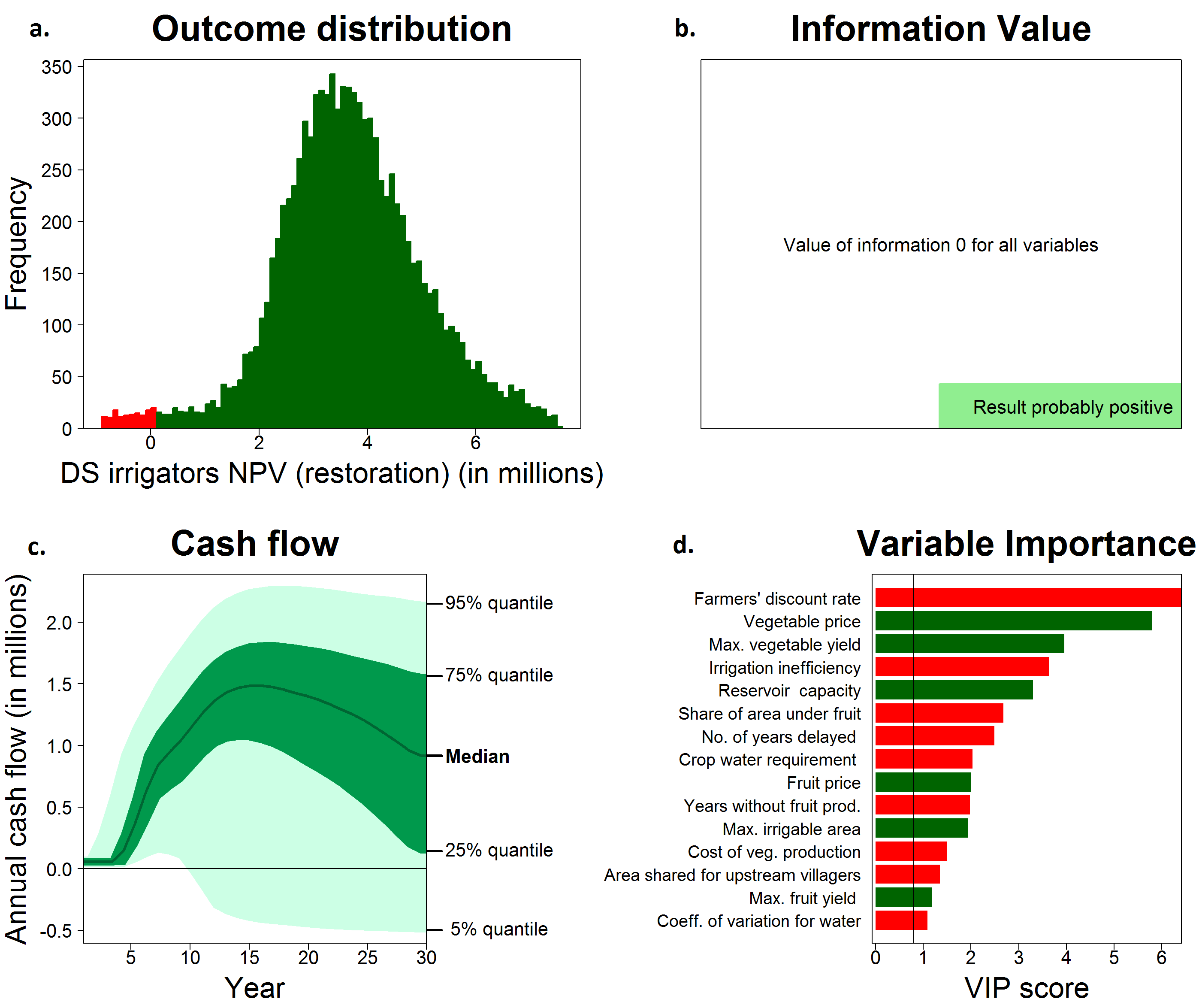


**Figure S7:** Simulation results from 10,000 model runs for the **downstream irrigators** for the implementation of a proposed irrigation dam, **with catchment restoration**, in Tigray, Ethiopia.

For detailed description of the graphs and bars, see legend to Figure S1


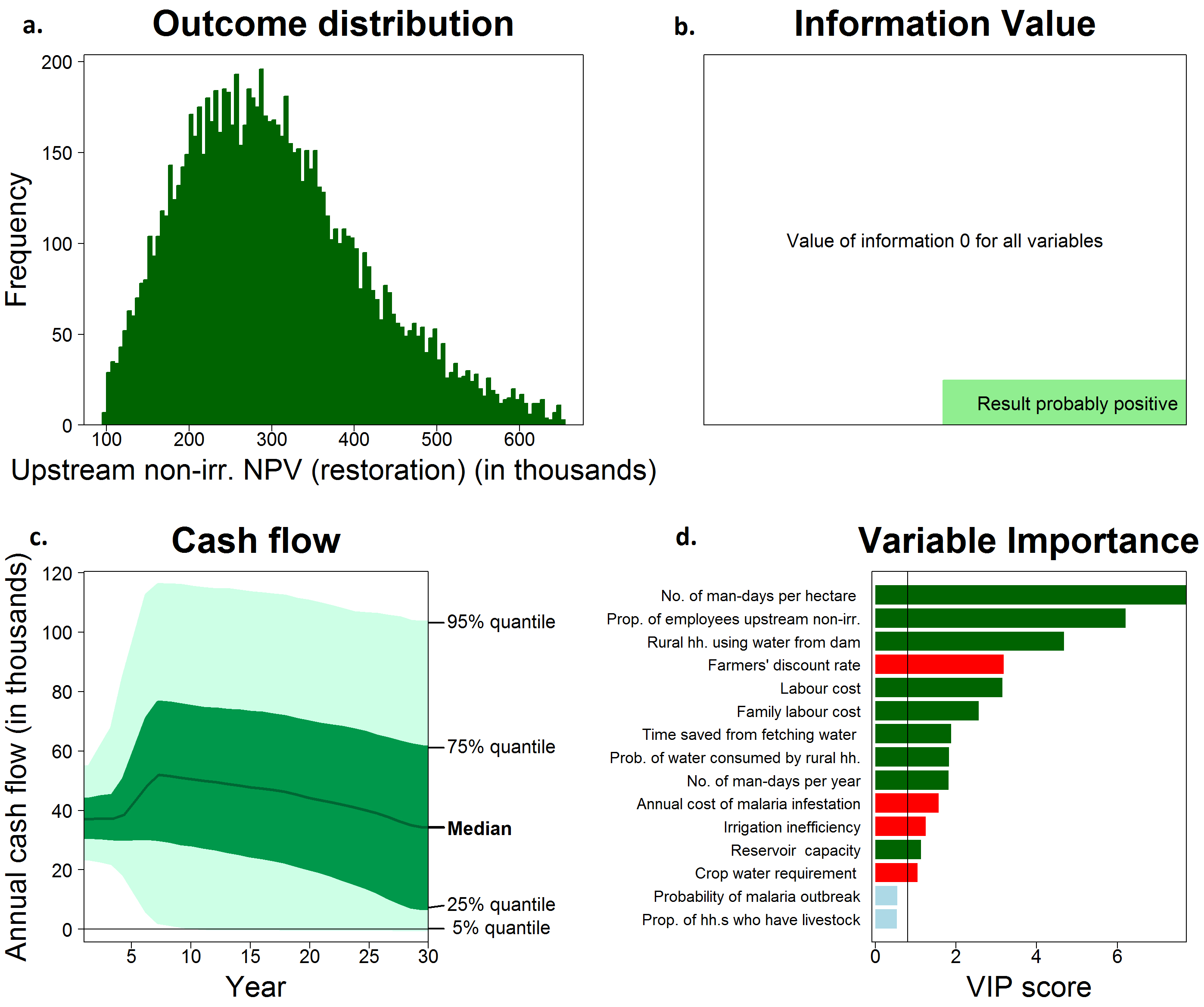


**Figure S8:** Simulation results from 10,000 model runs for the **upstream non-irrigators** for the implementation of a proposed irrigation dam, **with catchment restoration**, in Tigray, Ethiopia.

For detailed description of the graphs and bars, see legend to Figure S1


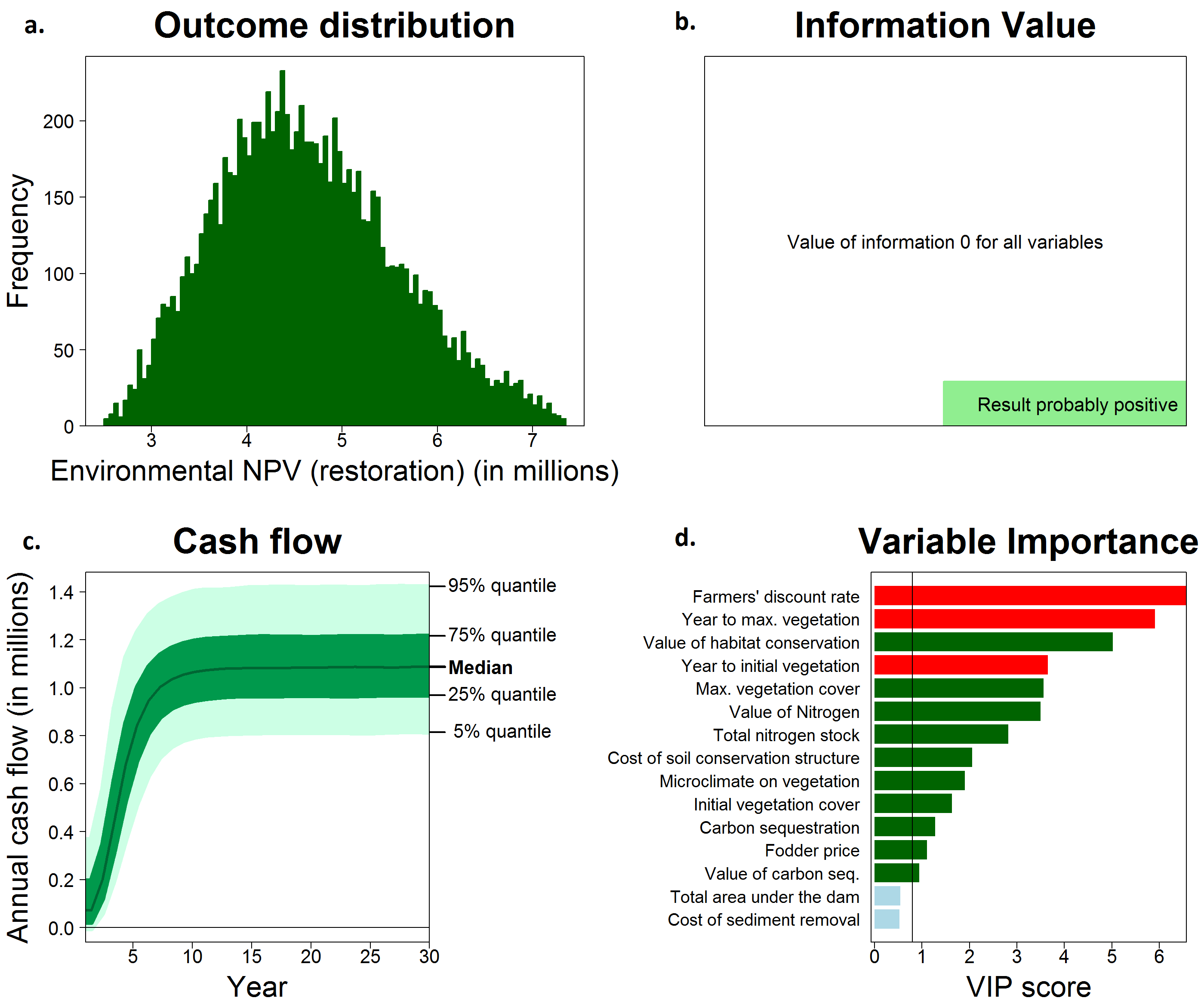


**Figure S9:** Simulation results from 10,000 model runs for the **environmental effects** of implementing an irrigation dam**, with catchment restoration**, in Tigray, Ethiopia.

For detailed description of the graphs and bars, see legend to Figure S1


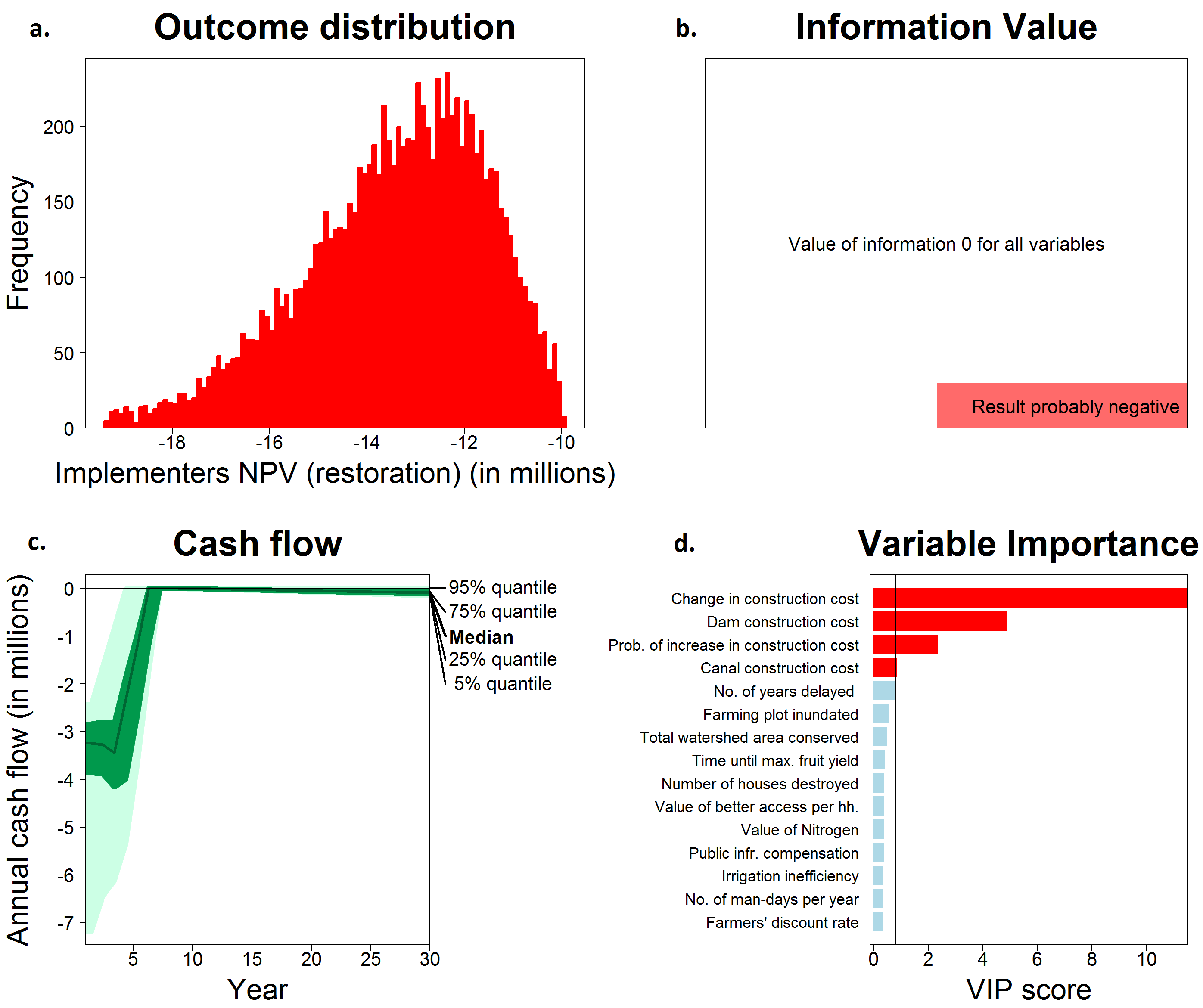


**Figure S10:** Simulation results from 10,000 model runs for the **implementer** for the implementation of a proposed irrigation dam, **with catchment restoration**, in Tigray, Ethiopia.

For detailed description of the graphs and bars, see legend to Figure S1

# **References**

Luedeling, E., Gassner, A., 2012. Partial Least Squares Regression for analyzing walnut phenology in California. Agric. For. Meteorol. 158–159, 43–52. https://doi.org/10.1016/j.agrformet.2011.10.020

Luedeling, E., Oord, A.L., Kiteme, B., Ogalleh, S., Malesu, M., Shepherd, K.D., De Leeuw, J., 2015. Fresh groundwater for Wajir—ex-ante assessment of uncertain benefits for multiple stakeholders in a water supply project in Northern Kenya. Front. Environ. Sci. 3, 1–18. https://doi.org/10.3389/fenvs.2015.00016

Tuffaha, H.W., Strong, M., Gordon, L.G., Scuffham, P.A., 2016. Efficient Value of Information Calculation Using a Nonparametric Regression Approach: An Applied Perspective. Value Heal. 19, 505–509. https://doi.org/10.1016/j.jval.2016.01.011

Wafula, J., Karimjee, Y., Tamba, Y., Malava, G., Muchiri, C., Koech, G., De Leeuw, J., Nyongesa, J., Shepherd, K., Luedeling, E., 2018. Probabilistic Assessment of Investment Options in Honey Value Chains in Lamu County, Kenya. Front. Appl. Math. Stat. 4, 1–11. https://doi.org/10.3389/fams.2018.00006

Whitney, C.W., Tabuti, J.R.S., Hensel, O., Yeh, C.H., 2017. Homegardens and the future of food and nutrition security in southwest Uganda. Agric. Syst. 154, 133–144. https://doi.org/10.1016/j.agsy.2017.03.009
